# Best Interests in the Mental Capacity Act: Time to say Goodbye? 

**DOI:** 10.1093/medlaw/fww030

**Published:** 2016-12-22

**Authors:** Mary Donnelly

**Affiliations:** School of Law, University College Cork, Ireland

**Keywords:** Autonomy, Best interests, Convention on the Rights of Persons with Disabilities, Court of Protection, Dignity, Impaired capacity, Will and preference

## Abstract

Article 12 of the United Nations Convention on the Rights of Persons with Disabilities, as interpreted by the Committee on the Rights of Persons with Disabilities in General Comment No. 1, offers a vision for law’s response to capacity impairments which differs in crucial ways from that contained in the Mental Capacity Act 2005. The Committee rejects the functional test for capacity and requires that a ‘will and preferences’ paradigm must replace the ‘best interests’ paradigm and that all substitute decision-making regimes must be abolished. This article draws on the position adopted in General Comment No. 1 in evaluating the best interests standard in the Mental Capacity Act. It sets out the normative case for a stronger legislative endorsement of will and preferences and the inclusion of greater support mechanisms but rejects the contention that all substitute decision-making can, or should, be abolished. It also argues that the best interests standard in the Mental Capacity Act retains some revolutionary potential and that, pending legislative reform, this can be further developed through the courts.

## 1. Introduction

Article 12 of the United Nations Convention on the Rights of Persons with Disabilities^1^ (CRPD), as interpreted by the Committee on the Rights of Persons with Disabilities,^2^ offers a vision for law’s response to capacity impairments which differs in crucial ways from that contained in the Mental Capacity Act 2005 (MCA). In General Comment No. 1 (2014, GC1),^3^ the Committee rejects both differential treatment on the basis of an application of a functional test for capacity^4^ and the use of the best interests standard for decision-making. Instead, it requires that ‘the “will and preferences” paradigm must replace the “best interests” paradigm to ensure that persons with disabilities enjoy the right to legal capacity on an equal basis with others.’^5^ Unfortunately, GC1 does not indicate precisely what is meant by either paradigm. However, it does make clear that, in the Committee’s view, compliance with Article 12 requires that States Parties abolish all substitute decision-making regimes.^6^ Thus, although the expanded best interests standard in the MCA, which requires account to be taken of the person’s wishes and feelings,^7^ shows more respect for individual subjectivity than prior iterations of the standard, it does not meet the requirements of Article 12, as interpreted by GC1.^8^

This article draws on the position put forward in GC1 as part of a broader normative evaluation of decision-making in situations of impaired capacity. It argues that the MCA should be strengthened by the inclusion of a stronger statement of the primary importance of the individual’s wishes and feelings and by the inclusion of enhanced support requirements and that the terminology of best interests should be replaced by a terminology of rights. However, it also argues that a substitute decision-making framework, openly recognised as such, should continue to operate alongside a supported decision-making framework and that such a framework should allow account to be taken of factors beyond will and preferences albeit in a restricted number of situations.

The article begins by contextualising GC1 within the values and goals of the CRPD. It then draws on the two grounding principles of autonomy and dignity to justify a paradigm which prioritises will and preferences but also to argue that an appropriate legal framework must do more than simply require adherence to will and preferences. This is followed by a critique of GC1’s fallback position of ‘best interpretation of will and preferences’ in situations in which a person’s will and preferences cannot be determined.^9^ The final section of the article examines how the wishes and feelings element of the MCA best interests test has been operating and explains why statutory amendment is needed to strengthen this aspect of the MCA. However, it also shows that, even without amendment, the MCA retains a degree of revolutionary potential and that this could (and should) be further developed.

## II. GENERAL COMMENT NO. 1 IN CONTEXT

The CRPD came into effect on 3 May 2008 and, at the time of writing, has been ratified by 159 countries/regional entities (although thirty-two countries/regional entities ratified subject to declarations or reservations).^10^ The drafters of the CRPD regarded the Convention as representing a ‘paradigm shift’,^11^ a new way of thinking about disability, centred on citizenship, equality, and inclusion. In this, the CRPD utilises the strengths of the social model of disability to provide a powerful manifesto for change. At the core of the social model is a distinction between impairment (a person’s underlying condition) and disability, which represents the way in which impairment has been socially constructed.^12^ The power of this distinction lies in the way it draws attention to the ways in which societal change can reduce or remove the impact of disability. Thus, as described by Oliver Lewis, the CRPD plays an ‘educative, expressive and proactive’ role.^13^

Notwithstanding the utility of its political message, aspects of the social model remain contentious within disability studies. While it is inarguably the case that oppressive conditions in society cause impairments to become disabilities, the social model has been criticised for failing to recognise and address the lived impact of impairment (as opposed to disability). Thus, it is argued that the model fails to take account of the realities of embodiment—ie people exist as bodies and experience disability both socially and corporeally.^14^ As yet, less work has been done from a disability studies perspective regarding the lived realities of mental impairments (what Margaret Price calls ‘bodymind’).^15^ This work is especially challenging in respect of people with profound capacity impairments. It is, however, essential in order to provide a convincing conceptual grounding for CRPD ideals.

By requiring that ‘persons with disabilities enjoy legal capacity on an equal basis with others in all aspects of life’,^16^ Article 12 represents the most conceptually challenging element of the CRPD paradigm shift.^17^ Unsurprisingly, its drafting was contentious^18^ and the compromises reached are reflected in the sometimes opaque language of the final article and in the (deliberate) omission of a specific reference to the permissibility or otherwise of substitute decision-making.^19^ Subsequently, commentators offered varying interpretations of Article 12, with several arguing that, notwithstanding the absence of an express prohibition, Article 12 in fact prohibited any form of substitute decision-making.^20^

This is also the approach taken by GC1, which on first sight appears to proceed on the basis that all decisions can be supported. However, GC1 does not follow through entirely on this position because it also acknowledges that situations can arise ‘where, after significant efforts have been made, it is not practicable to determine the will and preferences of an individual’.^21^ In such cases, GC1 states that the “‘best interpretation of will and preferences” must replace the “best interests” determinations’.^22^

## III. CHOOSING THE PARADIGM: NORMATIVE ARGUMENTS

Although GC1 is clear regarding the need to adopt a will and preferences paradigm, it is less explicit regarding what a legal framework based on such a paradigm would involve. However, GC1’s rejection of substitute decision-making, read alongside its statement that ‘at all times, including in crisis situations, the individual autonomy and capacity of persons with disabilities to make decisions must be respected’,^23^ suggests that there are no circumstances in which a decision which runs counter to a person’s will and preferences should be permitted.

The will and preferences paradigm should be understood in the context of decision-making supports required under Article 12(3) of the CRPD and the supported decision-making regime which GC1 outlines. However, GC1 does not link the operation of its will and preferences paradigm with a requirement that support has been provided in respect of a particular decision. In fact, GC1 states that some persons with disabilities may not wish to exercise their right to support.^24^

The normative discussion below proceeds on the basis of this understanding of GC1. The discussion focuses on the substantive principles of autonomy and dignity rather than equality/non-discrimination, which is also core to the CRPD.^25^ This is because equality/non-discrimination as a principle must incorporate external values, such as autonomy and dignity. As Peter Westen has argued ‘[w]ithout moral standards, equality remains meaningless, a formula that can have nothing to say about how we should act.’^26^ Thus, it is essential to understand how substantive principles apply rather than simply adopting an equality analysis.

### A. Respecting Will and Preference: The Normative Case in Favour

While at first sight, a simple focus on preference satisfaction could be seen to derive from a particularly ‘thin’ and ethically unconvincing^27^ form of autonomy, when taken together with the broader support requirements in Article 12(3), a justification based on a ‘thicker’ or more relational form of autonomy may be made.^28^ This justification is that capacity impairment is not necessarily a barrier to people having views and preferences (acting autonomously in a thin sense) or having values, life narratives and a sense of selfhood, and seeking to formulate ways in which to give effect to these (acting autonomously in a thicker sense).^29^ The fact that a person may need assistance or support in doing this does not, of itself, render their actions any less autonomous (in either the thinner or thicker sense).^30^

Respect for will and preferences regardless of capacity impairment may also be justified on dignity principles. Michael Rosen isolates ‘four strands in the conceptual make-up of dignity’.^31^ These are: dignity as rank or status; the Kantian conception of dignity as the intrinsic value of human beings; dignity as self-possessed behaviour; and, dignity as a requirement that people be treated with respect.^32^ The first and fourth of these strands are especially useful in the current context.^33^ Jeremy Waldron advances a view of dignity which keeps faith with ‘its ancient connection to noble rank or high office’.^34^ Crucially, however, he links this to an egalitarian theory of rights whereby ‘the modern notion of *human* dignity involves an upward equalization of rank, so that we now try to accord to every human being something of the dignity, rank, and expectation of respect that was formerly accorded to the nobility.’^35^ Explaining how this re-conceptualisation might work, Waldron suggests ‘[t]hink, for example, of the change that comes when one views an assault on an ordinary man or woman not just as a crude physical interference, but as a sort of sacrilege (like assaulting a prince or duke).’^36^

Applied in the current context, it can be argued that, just as the preferences of persons of high rank were once elevated by his or her servants and functionaries, we should now afford a similar level of respect to all persons’ preferences (regardless of capacity impairment). This view of dignity helps to explain why a person’s dignity may be violated not just by the steps required for the physical enactment of a decision taken contrary to his or her will (eg physical restraint or the use of psychological force), but also by inaction or indifference to his or her will and preferences. This is helpful because, while it is relatively straightforward to recognise how the use of force to impose one person’s will on another can be a violation of that other person’s dignity^37^ (as well as a violation of his or her bodily and psychological integrity), the dignitary harm done by indifference to a person’s will and preferences may not be so immediately obvious.

Describing the fourth strand, the conception of dignity as a requirement that people be treated with respect, Rosen argues that this understanding ‘emphasizes the value of the symbolic or expressive aspects of our behaviour toward others’.^38^ Respecting a person’s dignity ‘is both a matter of what we do and of how (that is to say, with what attitude) we do it’.^39^ Thus, this conception of dignity is concerned with process, as well as substance, and serves to reinforce the unacceptability of indifference to the will and preferences of a person with impaired capacity.

### B. Normative Limitations of the GC1 Will and Preferences Paradigm

While the case for respect for will and preferences may be grounded in both autonomy and dignity principles, this case does not apply universally, as the GC1 will and preferences paradigm appears to require. While autonomy principles underpin the right to decision-making support (both generally and in respect of individual decisions), respect for autonomy can only justify respect for will and preferences in respect of an individual decision where the conditions for an autonomous decision have been met in respect of the decision in question. This is not recognised in GC1 which appears to operate on the basis that if the Article 12(3) requirement for the provision of decision-making support has been met, autonomous decisions will follow. There are two difficulties with this. First, leaving aside the matter of whether, in principle, everyone can be supported to make a decision,^40^ across the range of people who require support because of impaired capacity, unavoidably support will sometimes be inadequate. What constitutes an adequate support depends on the conception of autonomy adopted.^41^ However, even with a relatively thin conception of autonomy, it is difficult to envisage how, at least in the context of decisions with significant consequences, support could be considered sufficient if it does not involve some degree of development of capabilities for self-reflection,^42^ so that people can make their significant decisions in the context of their own broader life narrative.

The difficulty in this regard, as Terry Carney and Fleur Beaupert argue, is that there is no reason to believe that the provision of decision-making supports would be exempt from the ‘divergence between the aspirations of policymakers keen to expand personal autonomy of action and personalised decision-making, and the harsh realities of actual experience’ which has been clearly evident in similar areas.^43^ Support provided will be variable in quality, whether because of inadequate financial resources dedicated by the State or because of the vagaries of the humans who provide the support. Moreover, as Michael Bach and Lana Kerzner recognise, decision-making support is better understood as a process rather than an event, requiring the development of relationships of trust.^44^ In relation to some decisions, it will not be possible in advance of the decision to be made to develop the relationships necessary to provide the required degree of support.

The second problem does not necessarily derive from the existence of capacity impairment,^45^ although it may be exacerbated by this. This relates to the impediments to agency, whether arising through the actions of others such as undue influence, coercion or manipulation, or through societal or structural contexts which reduce or remove an individual’s potential for autonomous decision-making. These impediments mean that conditions for autonomy (even on a minimalist interpretation) will not always be met in the context of individual decisions.^46^ Thus, while respect for autonomy grounds the right of support and a right to respect for will and preferences, it does not ground either a legal framework based exclusively on respect for will and preference or an absolute abolition of substitute decision-making.

A similar limitation emerges in the context of the grounding in dignity principles. Although respect for dignity presumptively requires respect for autonomy, as Mary Neal notes, ‘[a]utonomy is far from being *all* there is to dignity: respecting dignity will not always involve respecting autonomy and conversely, it is conceivable that someone might respect autonomy … while failing to respect other aspects of human dignity.’ ^47^ Regardless of how dignity is conceptualised, it involves objective as well as subjective elements.^48^ Respect for a person’s dignity generates, in Waldron’s words, ‘a duty to respect that person (in various ways)’.^49^ It recognises that the ‘perils of indifference’^50^ operate in two directions. Just as indifferent disregard for will and preferences fails to respect a person’s dignity, an indifferent accession to will and preferences may also fail to respect his or her dignity. On this understanding of dignity, sometimes it may be justifiable to do something which is not in accordance with a person’s will and preferences. If we take the example of a person living in conditions of extreme self-neglect who rejects all offers of assistance and refuses all attempts at communication, we can see that his or her dignity (as well as several of his or her rights^51^) may be compromised. In such circumstances, intervention is not only consistent with respect for dignity; it is required.^52^ In this context, respect for dignity retains its significance. Indeed, as Rosen identifies, dignity assumes particular importance in situations where ‘we consider it right to treat people in ways that go drastically against the ways in which they themselves wish to be treated’.^53^ Rosen argues that in these situations ‘it is of great importance that we act in ways that express—perhaps to them, and perhaps to others—that they are acknowledged as having the entitlement to be treated with respect.’^54^ Ultimately, as with autonomy, dignity principles ground respect for will and preferences (as well as other respect requirements) but they do not ground the will and preferences paradigm as advanced in GC1 or the abolition of substitute decision-making in all circumstances.

### C. Best Interpretation: The Fallback Position

GC1 adopts a fallback position, based on the ‘best interpretation of will and preferences’, which is to apply where, following significant efforts, a person’s will and preferences cannot be determined. Because this approach permits the hidden exercise of power, it is argued here that it is not an appropriate fallback standard and should not be adopted.

GC1 does not provide any detail on how a ‘best interpretation’ approach might operate,^55^ although it does require the provision of non-discriminatory advance planning mechanisms,^56^ which would be of assistance if the person had availed of these. In the absence of more formalised advance planning, established prior preferences, values, attitudes, and narratives may be important indicators of what a person’s current will and preferences might be. The fact that a person had impaired capacity is no barrier to drawing on these indicators, so long as the indicators may be identified. Thus, sometimes, best interpretation could operate broadly along the lines of the substituted judgment standard for decision-making which has been applicable in the United States since the 1970s.^57^ The difficulty, as rapidly became apparent with the substituted judgment standard,^58^ is that sometimes there will be no indication of what a person’s will and preferences in respect of the particular decision at issue would be. For more straightforward decisions, this need not necessarily be problematic because it can reasonably be presumed that most people who do not express a contrary view would prefer actions which enhance rather than diminish their broader well-being. Thus, if no other information is available, we might reasonably apply the best interpretation standard to interpret a person’s preference as wishing to be warm, comfortable, well-nourished, cared-for, and treated with respect.

However, not all decisions are straightforward and in these situations, a best interpretation standard becomes problematic. Even presuming that the person who is interpreting will and preferences fits (insofar as humans can)^59^ within Eva Kittay’s ideal of the ‘transparent caregiver’, who separates his or her own needs from those of a cared-for person,^60^ there are evident risks in constructing a narrative about another person.^61^ Most immediately, we may get the interpretation wrong. An example provided by Kittay relates how both she, as a loving mother, and Peggy, as a long-time, devoted, professional carer, struggle to discern the needs of her daughter, who has profound capacity impairments:

When Sesha is ill, we don’t know what bothers her, what hurts her, what the pain feels like. We are deprived of a vital avenue for diagnosis. This makes her so vulnerable, and makes us crazy.^62^

The problem is that the best interpretation standard refuses to acknowledge that there are things that we do not, and cannot, know. It forgoes epistemic humility and assumes levels of knowledge (and justifications for actions) in situations where they do not exist. And, given that the people about whom these decisions are being made will not be in position to argue otherwise, this can happen without fear of contradiction.

If it were translated into a legal standard, a best interpretation approach would require those charged with making decisions to engage, in some circumstances at least, in what Guido Calabresi describes as ‘legal subterfuge’.^63^ Calabresi notes decision-makers’ preference for legal subterfuges in the context of ‘tragic choices’,^64^ where their employment allows decision-makers to avoid having to identify and articulate the normative basis for a decision. The problem with legal subterfuges is that, as was recognised by Jeremy Bentham more than 200 years ago, when law parts company with reality, the exercise of power becomes obscured.^65^ As described by Louise Harmon, in the context of the operation of the substituted judgment standard in the United States, ‘[s]omething hidden, something potentially dangerous or brutal, can go on beneath the surface’.^66^ It is for this reason that the law must continue to acknowledge that some of the time, and inevitably, decisions will be made for people and that sometimes these decisions cannot be made on the basis of their will and preferences. Where decisions concern people whose capacity to assert their interests is profoundly reduced, the risks of any form of subterfuge are too significant to allow for anything other than an overt recognition of when and how power is being exercised and the maintenance of rigorous oversight of such exercise.^67^

## IV. WISHES AND FEELINGS IN THE MCA

In its own way, the incorporation of a person’s ‘past and present wishes and feelings’ into the MCA best interests standard represented something of a paradigm shift.^68^ While the terminology of respect for wishes and feelings is different, this requirement is, in essence, similar to the CRPD requirement for respect for will and preferences. Indeed, the MCA formulation is preferable, because of its more overt recognition of the role of affective elements in decision-making.^69^ The MCA also includes the beginnings of a support model, requiring that the person in respect of whom a best interests determination is being made should be permitted and encouraged to participate in the process and that his or her ability to participate should be improved^70^ and requiring consultation with relevant others in order to determine the person’s best interests and his or her wishes and feelings.^71^ The support element is further enhanced by the provision made for the appointment of Independent Mental Capacity Advocates (IMCAs) to provide support in designated circumstances.^72^ However, the support model is limited not least because its sole focus is on supporting participation in individual decisions but rather on developing an individual’s autonomy in a broader and more profound sense.

Perhaps because of the innovative nature of the provision, the MCA is unforthcoming regarding the weighting to be afforded to wishes and feelings when compared with the other elements of the best interests standard. It is unsurprising, therefore, that the wishes and feelings element has been inconsistently applied by the courts.^73^ The decision of Munby J. in *ITW v Z* provides the most closely worked analysis of the relevant requirement.^74^ Munby J. acknowledges that wishes and feelings ‘will always be a significant factor to which the court must play close regard’.^75^ However, the force of this assertion is diminished by the subsequent statement that the weight to be attached to wishes and feelings will ‘always be case-specific and fact-specific’.^76^ Its impact is also reduced by Munby J.’s identification as a crucial factor, the extent to which the person’s wishes and feelings, if given effect to, ‘can properly be accommodated within the court’s overall assessment of what is in [the person’s] best interests’.^77^ This approach does not recognise the profundity of the challenge which respect for wishes and feelings can pose for the objective best interests standard and the extent to which the determination of what is in a person’s best interests is altered once his or her wishes and feelings are taken into account.

Notwithstanding these limitations, Munby J.’s checklist has provided an important framework for best interests decision-making. The application of the checklist in *Westminster City Council v Manuela Sykes*^78^ shows both the potential of this aspect of the MCA and also why a simple preference satisfaction paradigm would be inadequate in practice. In this case, District Judge Eldergill found that it was in the best interests of Ms Sykes, who had impaired capacity because of dementia, to return to her flat (where she had lived for 60 years)^79^ on a 1-month trial basis, notwithstanding that the local authority, the independent psychiatrist, Ms Sykes’ nieces and her religious advisor all considered it, on balance, to be in her best interests to remain in care. In so finding, District Judge Eldergill recognised Ms Sykes’ consistent wish was to live in her home for as long as possible.^80^ Acknowledging that, if a trial return home was not attempted at this time, Ms Sykes would ‘never again have the opportunity to live in her own home’,^81^ District Judge Eldergill found that ‘the fact that significant problems and some distress lie ahead, and that “failure” is quite likely’ should not prevent Ms Sykes from having the opportunity to attempt some last months of freedom in her own home at the end of her life.

Perhaps the most instructive feature of this case is the way in which the court, working with the local authority and RS, Ms Sykes’ elderly and ‘heroic’ carer, sought a workable solution, which facilitated, insofar as was possible, acting in accordance with Ms Sykes’ wishes and feelings. As District Judge Eldergill acknowledged, the solution was likely to be a temporary one. Nonetheless, by placing Ms Sykes’ wishes and feelings at the centre of the effort to reach a solution, the Court recognised and respected her inherent right to dignity. The working through of a solution within the MCA framework also meant that factors, such as Ms Sykes’ limited finances and the strain on RS, could be openly addressed and that the realities of decision-making in the context of capacity impairment were not buried in legal subterfuge. *Sykes* is an example of emerging jurisprudence of the Court of Protection^82^ which, read together with the reassertion of the significance of wishes and feelings by the Supreme Court in *Aintree University Hospitals NHS Trust v James,*^83^ provides the basis for a more expansive, arguably revolutionary, view of best interests under the MCA.

There is also, however, an alternative line of jurisprudence which accords much less weight to the wishes and feelings element of the MCA standard. The most notable recent decision is that of the Court of Appeal in *RB v Brighton and Hove City Council.*^84^ Here, the Court of Appeal found that a deprivation of RB’s liberty was necessary ‘in order to protect RB from seriously injuring himself’ and that [‘t]hat must be in his best interests’.^85^ The fact that RB had expressed a consistent wish to live in the community was dismissed by the Court on that basis that ‘[u]nfortunately it is not possible for the time being to comply with those wishes.’^86^ The approach taken suggests indifference to RB’s wishes and feelings and this is reinforced by the absence of any attempt to consider/mandate the consideration of whether a workable solution could be reached. While clearly the Court of Appeal is not suited to the kind of fine-grained analysis which can be undertaken in lower courts*,* it is unfortunate that the Court missed the opportunity to lend its support, in principle, to this kind of approach.

Decisions such as *RB* strengthen the case for an amendment of the MCA so as to include a legislative requirement for a greater level of respect to be afforded to the wishes and feelings of the individual^87^ and to broaden the scope for decision-makers to investigate appropriate solutions.^88^ Moreover, respect for autonomy (as well as compliance with Article 12(3) of the CRPD) requires amendment of the MCA to enhance supported decision-making frameworks.^89^

Alongside these substantive amendments, a case may be made that it is time for a change in terminology. As a term, ‘best interests’ has some advantages, including that it gives judges and other decision-makers a useful shorthand to signify the absolute primacy of the person.^90^ However, since the MCA, this term has been required to signify something quite different to its original meaning.^91^ There is, therefore, a dissonance between the original, objective, meaning of the term and its current meaning under the MCA and this dissonance would be accentuated by the substantive amendments proposed here. Although best interests is, to a degree, an indeterminate term,^92^ capable of assimilating shifts in meaning, there are still difficulties with retaining the same term but hoping that it will serve as a shorthand for something different. This is because decision-makers’ intuitions associated with the old meaning will continue to assert themselves with the result that the underlying conceptual shift cannot be delivered upon.^93^

For the reasons argued above, replacing the shorthand of best interests with the shorthand of will and preferences would be overly simplistic and misleading. One alternative, adopted in the Assisted Decision-Making (Capacity) Act 2015 (Ireland), is to replace the term best interests with a set of applicable principles which reflect the more complex nature of decision-making.^94^ However, the Act runs into difficulties when attempting to find a shorthand expression of the central importance of the individual and has fallen back on the (unsatisfactory) shorthand term ‘benefit’.^95^ The inclusion of an expanded set of principles in the MCA could be useful but a shorthand is still needed. A more suitable alternative could draw on the terminology of rights, ie all actions must respect the rights of the person. Although this terminology is in many ways as indeterminate as that of best interests, its adoption could help realign decision-making frameworks to afford greater recognition to a person’s will and preferences while also recognising the more complex interplay of factors at play.

## V. CONCLUSION

The CRPD aims to change the way in which law and policymakers respond to capacity impairment so that inclusion, participation, and citizenship are normalised. In this new environment, it is appropriate to revisit the conceptual underpinnings for the MCA best interests standard. Drawing on the principles of autonomy and dignity, this article has evaluated the MCA standard and the alternative vision put forward by the Committee on the Rights of Persons with Disabilities in GC1. Although it has concluded that some elements of the will and preferences paradigm put forward by GC1 are not normatively justified, it has also argued, based on these principles, that the will and preferences/wishes and feelings of a person with impaired capacity should receive a higher degree of respect than has sometimes been the case under the MCA and that the terminology of best interests should be replaced with a terminology of rights. While legislative amendment would be required to deliver on some of the proposals made here, the enhancement of respect for wishes and feelings can be (and in some cases is being) achieved within the current MCA best interests standard. Although, as acknowledged by District Judge Eldergill in *Sykes*, in the messy, lived world where decisions have to be made, there are no perfect solutions,^96^ the MCA’s potential to provide a legal framework for principled, open, and accountable decision-making should not be underestimated.

## Footnotes

1 A/RES/61/106 Annex 1.

2 Although interpretations of UN Conventions in General Comments are not legally binding, they can be influential in developing policy positions, in particular where there is a consensus as to their normative legitimacy: see H Keller and L Grover, ‘General Comments of the Human Rights Committee and their Legitimacy’ in H Keller, G Ulfstein, L Grover (eds), *UN Human Rights Treaty Bodies: Law and Legitimacy* (CUP 2012) 128–29.

3 CRPD/C/GC/1.

4 GC1, para 15.

5 ibid, para 21.

6 ibid, para 28. GC1 states (para 27) that substitute regimes may take different forms but they share the characteristics that: ‘(i) legal capacity is removed from a person, even if this is in respect of a single decision; (ii) a substitute decision-maker can be appointed by someone other than the person concerned, and this can be done against his or her will; and (iii) any decision made by a substitute decision-maker is based on what is believed to be in “objective” best interests of the person concern, as opposed to being based on the person’s own will and preferences.’

7 MCA, s 4(6).

8 This is also the view of the Essex Autonomy Project, *Achieving CRPD Compliance: Is the Mental Capacity Act of England and Wales Compatible with the UN Convention on the Rights of Persons with Disabilities? If Not, What Next?* (Report to the UK Ministry of Justice, September 2014); for similar conclusion, see R Harding, ‘The Rise of Statutory Wills and the Limits of Best Interests Decision-Making in Inheritance’ (2015) 78 MLR 945, 967.

9 GC1, para 21.

10 Although the UK has included both reservations and declarations, none relate to the matter discussed here. However, several Member States (Australia, Canada, and Norway) have issued interpretative declarations that it is their understanding that the CRPD allows for substitute decision-making where such arrangements are necessary, as a last resort and subject to safeguards. Ireland has also indicated its intention to ratify subject to this declaration: see Roadmap to Ratification of the United Nations Convention on Persons with Disabilities <http://www.justice.ie/en/JELR/Pages/PB15000549> accessed 20 July 2016.

11 See the statement of Don MacKay, the Chairman of the Ad Hoc Committee which developed the text of the CRPD in ‘High Level Dialogue: From Vision to Action: The Road to Implementation of the Convention on the Rights of Persons with Disabilities’ (New York, 30 March 2007) <http://www.un.org/disabilities/convention/signature.shtml> accessed 20 July 2016.

12 This is contrasted with a conceptualisation of disability as an individual affliction (sometimes described as the medical model): see R Kayess and P French, ‘Out of Darkness into Light? Introducing the Convention on the Rights of Persons with Disabilities’ (2008) 8 HRLR 1, 5–12; B Clough, ‘“People Like That”: Realising the Social Model in Mental Capacity Jurisprudence’ (2014) 23 Med L Rev 53.

13 O Lewis, ‘The Expressive, Educational and Proactive Roles of Human Rights: An Analysis of the United Nations Convention on the Rights of Persons with Disabilities’ in B McSherry and P Weller (eds), *Rethinking Rights-Based Mental Health Law* (Hart Publishing 2010) 98.

14 See eg L Crow, ‘Including all of our Lives: Renewing the Social Model of Disability’ in J Morris (ed), *Encounters with Strangers: Feminism and Disability* (Women’s Press 1996); T Shakespeare, *Disability Rights and Wrongs* (Routledge 2006); A Kafer, *Feminist, Queer, Crip* (Indiana UP 2013).

15 M Price, ‘The Bodymind Problem and the Possibilities of Pain’ (2015) 30 Hypatia 268, 268.

16 art 12(2).

17 See G Quinn, ‘Personhood and Legal Capacity: Perspectives on the Paradigm Shift of Article 12 CRPD’ (Conference on Disability and Legal Capacity under the CRPD, Harvard Law School, 20 February 2010) <http://www.inclusionireland.ie/content/page/documents-and-reports-3rd-parties> accessed 20 July 2016.

18 See A Dhanda, ‘Legal Capacity in the Disability Rights Convention: Stranglehold of the Past or Lodestar for the Future?’ (2006–2007) 34 Syracuse Jl Int L & Com 429, 438–56.

19 On the context for this omission, see ibid 448.

20 See T Minkowitz, ‘United Nations Convention on the Rights of Persons with Disabilities and the Right to be Free from Nonconsensual Psychiatric Interventions’ (2006–2007) 34 Syracuse Jl Int L & Com 405, 408–11; E Flynn and A Arstein-Kerslake, ‘Legislating Personhood: Realising the Right to Support in Exercising Legal Capacity’ (2014) 10 Intl JLC 81, 89–91; E Flynn and A Arstein-Kerslake, ‘Support Model of Legal Capacity: Fact, Fiction or Fantasy?’ (2014) 32 Berk JIL 124, 132.

21 GC1, para 21.

22 ibid.

23 ibid, para 18.

24 ibid, para 19.

25 ibid, para 4.

26 P Westen, ‘The Empty Idea of Equality’ (1982) 95(3) Har L Rev 537, 547.

27 This is caricatured by Margaret Brazier as a demand that ‘I must be given what I want.’: M Brazier, ‘Do No Harm – Do Patients Have Responsibilities Too?’ (2006) 65 CLJ 397, 400; see also critiques by D Callahan, ‘Individual Good and Common Good: A Communitarian Approach to Bioethics’ (2003) 46 Perspect Biol Med 496; O O’Neill, *Autonomy and Trust in Bioethics* (CUP 2002).

28 Although there are various accounts of relational autonomy, a useful unifying concept, as advanced by John Christman, ‘Relational Autonomy, Liberal Individualism and the Social Construction of Selves’ (2004) 117 Phil Stud 143, 147 is that what makes a conception of autonomy uniquely relational is that ‘among its defining conditions are requirements concerning the interpersonal or social environment of the agent’.

29 An ability to act ‘out of genuine preference or character or conviction or sense of self’ is fundamental to Ronald Dworkin’s liberal account of autonomy: see *Life’s Dominion: An Argument about Abortion, Euthanasia and Individual Freedom* (Alfred A. Knopf 1993) 225.

30 See L Francis and A Silvers, ‘Thinking about the Good: Reconfiguring Liberal Metaphysics (or Not) for People with Cognitive Disabilities’ in E Kittay and L Carlson, *Cognitive Disability and its Challenge to Moral Philosophy* (Wiley-Blackwell 2010).

31 M Rosen, *Dignity: Its History and Meaning* (Harvard UP 2012) 114.

32 ibid.

33 While elements of the Kantian conception are also useful (in particular the egalitarian elements: on which, see Rosen (ibid 31), the linkage with rationality and the rational choosing of one’s moral law (on which, see ibid 30) makes the Kantian conception of dignity more problematic as a foundation.

34 J Waldron, *Dignity, Rank and Rights: The Berkeley Tanner Lectures* (OUP 2012) 30.

35 ibid 33 (original emphasis).

36 ibid 34.

37 This was recognised by the European Court of Human Rights in *Herczegfalvy v Austria* (1992) 15 EHRR 437 in respect of forced feeding and forced medication (albeit that the Court also found (para 82) that ‘as a general rule, a measure which is a therapeutic necessity cannot be regarded as inhuman or degrading’ within the meaning of art 3 of the European Convention on Human Rights).

38 Rosen (n 31) 126.

39 ibid.

40 Although examples are advanced of supported decision-making even in circumstances of quite extreme capacity impairment: see Flynn and Arstein-Kerslake, ‘Legislating Personhood’ (n 20) 100–01, there is a need for a good deal more rigorous empirical work before reaching definitive conclusions regarding the scope of what is possible in this regard.

41 See Ronald Dworkin’s (classic liberal) description of the capabilities needed for autonomous action (n 29); compare the more relational approach of Diana T Meyers, *Self, Society and Personal Choice* (Columbia UP 1989) 76, who argues that ‘[a]utonomous people must be able to pose and answer the question “What do I really want, need, care about, believe, value etcetera?”; they must be able to act on the answer; and they must be able to correct themselves when they get the answer wrong.’

42 See the three forms of support outlined in M Bach and L Kerzner, *A New Paradigm for Protecting Autonomy and the Right to Legal Capacity* (Law Reform Commission of Ontario 2010) 73.

43 T Carney and F Beaupert, ‘Public and Private Bricolage: Challenges Balancing Law. Services and Civil Society in Advancing CRPD Supported Decision-Making’ (2013) 36 UNSWLJ 175, 193–94.

44 Bach and Kerzner (n 42) 24.

45 See P Gooding, ‘Navigating the “Flashing Amber Lights” of the Right to Legal Capacity in the United Nations Convention on the Rights of Persons with Disabilities: Responding to Major Concerns’ (2015) 15 HRLR 45, 56.

46 Although GC1 recognises the risk that undue influence may be exacerbated for people ‘who rely on the support of others to make decisions’, its primary focus is on ensuring that protection against undue influence ‘must respect the rights, will and preferences of the person, including the right to take risks and make mistakes’: GC1, para 22. Note also the narrow conceptualisation of undue influence, with undue influence being characterised as occurring where the quality of the interaction includes ‘signs of fear, aggression, threat, deception or manipulation’: para 22.

47 M Neal, ‘Respect for Human Dignity as “Substantive Basic Norm’” (2014) 10 Intl JLC 26, 41.

48 See eg conceptualisation of dignity as ‘objective human thriving’ in C Foster, *Human Dignity in Bioethics and Law* (Hart Publishing 2011). Note Foster’s argument (ibid 14) that this means that ‘[a]ny coherent exposition of dignity will be a communitarian exposition.’ For an alternative conceptualisation, also involving an objective element, see D Feldman, ‘Human Dignity as a Legal Value: Part 1’ [1999] Pub L 682, 687 as the respect due to ‘fellow creatures inhabiting a common moral universe’.

49 Waldron (n 34) 142.

50 The term is borrowed from Elie Wiesel’s speech ‘The Perils of Indifference’ delivered at the White House on 12 April 1999.

51 These include rights protected under the CRPD: the right to life (art 10); the right to protection from all forms of exploitation, violence, and abuse (art 16); the right to the highest attainable standard of health (art 25); and the right to an adequate standard of living and social protection (art 28).

52 Although some elements of this argument may be seen as similar to those of the Conseil d’Etat in the famous ‘dwarf-throwing’ case (where the Conseil upheld a prohibition on dwarf-throwing competitions on the basis of respect for dignity notwithstanding that the applicant (the, even person who would have been thrown) was a consensual enthusiastic, participant (for summary of the case, see Rosen (n 31) 63–67)), the example is deliberately chosen to indicate the need for a much more demanding standard before non-consensual intervention on the basis of protection of dignity interests could be justified.

53 Rosen (n 31) 127. Note that Rosen’s examples relate to fighting against people in a war or punishing them as part of the criminal justice system as opposed to acting to prevent a violation of dignity.

54 ibid.

55 Flynn and Arstein-Kerslake, ‘Legislating Personhood’ (n 20) 94 suggests that this approach requires attempts to be made to ‘draw out the imagined will and preferences of the person’.

56 GC 1, para 17.

57 This standard emerged following the decision of the Supreme Court of New Jersey in *Re Quinlan* (1976) 70 NJ 10, 70 that the appropriate question in determining whether to withdraw life-sustaining treatment was what the patient would have wanted: for summary, see M Donnelly, *Healthcare Decision-making and the Law: Autonomy, Capacity and the Limits of Liberalism* (CUP 2010) 185–92.

58 See L Harmon, ‘Falling off the Vine: Legal Fictions and the Doctrine of Substituted Judgment’ (1990) 100 Yale LJ 1, 36–54.

59 As J Herring recognises, ‘Losing It? Losing What? The Law and Dementia’ (2009) 21 CFLQ 3, 27, within any caring relationship, by necessity there is ‘give and take’ and the interests of the carer and the cared for person cannot be disentangled.

60 EF Kittay, *Love’s Labor: Essays on Women, Equality and Dependency* (Routledge 1999) 52.

61 On the challenges of constructing narratives around a person who does not use words, see S Clifford Simplican, ‘Care, Disability and Violence: Theorizing Complex Dependency in Eva Kittay and Judith Butler’ (2015) 30 Hypatia 217, 226–27.

62 Kittay (n 60) 159. Note also the range of interpretations (among caring family members/professional carers) to the responses of Peter (who had profound capacity impairments), to a floating helium balloon, described in J Porter and others, ‘Interpreting the Communication of People with Profound and Multiple Learning Difficulties’ (2001) 29 Brit J Learn Disabil 175 and the difficulties of families in interpreting the wishes of loved ones regarding treatment options in the context of a terminal diagnosis, reported in Sulmasy and others, *‘*The Accuracy of Substituted Judgements in Patients with Terminal Diagnosis’ (1998) 128 Ann Intern Med 621.

63 *Ideals, Beliefs, Attitudes and the Law* (Syracuse UP 1985) 88. A legal subterfuge differs from a legal fiction in that legal subterfuges do not provide the basis for the creation of legal rules, but ‘rather are deployed to mask the choices implicit in the application of existing legal rules’: P Smith, ‘New Legal Fictions’ (2007) 95 Georgetown LJ 1435, 1471.

64 Calabresi, ibid, 88 describes these as situations involving a clash of beliefs, often in a life-or-death context such as withdrawal of life-sustaining treatment or abortion.

65 See, in particular, J Bentham, Preface to *A Comment on the Commentaries and a Fragment on Government* as cited in Smith (n 63) 1466.

66 Herring (n 59) 70.

67 For this reason, it is also difficult to see how a best interpretation framework could meet the requirement in art 12 (4) of the CRPD that States Parties ensure that ‘all measures that relate to the exercise of legal capacity provide for appropriate and effective safeguards to prevent abuse’.

68 There is very limited evidence in pre-MCA case law of any role for the contemporaneous views of the person about whom a best interests determination was being made: see M Donnelly, ‘Best Interests, Patient Participation and the Mental Capacity Act 2005’ (2009) 17 Med L Rev 1, 5–6.

69 On the importance of emotion to decision-making, see A Damasio, *Descartes Error: Emotion, Reason and the Human Brain* (Avon Book 1994) and A Damasio, *The Feeling of What Happens: Body and Emotion in the Making of Consciousness* (Vintage 2000).

70 MCA, s 4(4). See also ch 3 of the MCA 2005 Code of Practice (Department of Constitutional Affairs, 2007).

71 ibid, s 4(7).

72 ibid, s 35. Note however, that the IMCA framework does not seem to have been particularly effective in assisting people to challenge determinations regarding what was in their best interests: see E Flynn, *Disabled Justice: Access to Justice and the UN Convention on the Rights of Persons with Disabilities* (Ashgate 2015) 98–99.

73 See A Ruck Keene and C Auckland, ‘More Presumptions Please? Wishes, Feelings and Best Interests Decision-Making’ [2015] Eld LJ 293.

74 [2009] EWHC 2525 (Fam) [35].

75 ibid [35].

76 ibid [35].

77 ibid [35]. The other relevant factors were the degree of incapacity; the strength and consistency of the views expressed; and the possible impact on the person of knowing that his or her wishes and feelings had not been respected.

78 [2014] EWCOP B9.

79 Ms Sykes was 89 years and had been compulsorily admitted to hospital under the Mental Health Act 1983 in October 2012 and subsequently moved to a nursing home. At the time of her admission, there were issues relating to a lack of acceptance of care, altercations with neighbours, self-neglect, unhygienic living conditions, weight loss, wandering, and a lack of awareness of dangers to her personal safety.

80 Ms Sykes had made a Lasting Power of Attorney with instructions to this effect and this clearly remained her view.

81 [2014] EWCOP B9, § 10.

82 See also the judgment of Peter Jackson J in *Re M (Best Interests: Deprivation of Liberty)* [2013] EWCOP 3456 and the decisions of District Judge Eldergill in *A London Local Authority v JH and MH* [2011] EWCOP 2420 and *HT v CK and ALA* [2012] EWCOP 4160.

83 [2013] UKSC 67 [45].

84 [2014] EWCA Civ 561. See also *W v M* [2011] EWHC 2443 (Fam); *The Mental Health Trust and Ors v DD and Anor* [2014] EWCOP 11.

85 ibid [83].

86 ibid [81].

87 An amendment along these lines is also advocated by the Law Commission, *Mental Capacity and Deprivation of Liberty: A Consultation Paper* Consultation Paper No 222 (Stationary Office, 2015) para 12.47; Essex Autonomy Project (n 8) 51–52; Ruck-Keene and Auckland (n 73) 300.

88 In this respect, there may also be an argument for reconsideration of the question of what kind of decision-making environment works most effectively in this regard, including an examination of the potential of multidisciplinary tribunal hearings, at least at first tier stage.

89 See eg the recommendations made in the Australian Law Reform Commission Report 124, *Equality, Capacity and Disability in Commonwealth Laws* (2014).

90 See the definition of best interests in A Buchanan and D Brock, *Deciding for Others: The Ethics of Surrogate Decision-Making* (CUP 1989) 88 as ‘acting so as to promote maximally the good of the individual’.

91 Although it sometimes seems as if the term best interests has been around for centuries, in fact, in the exercise of the *parens patriae* jurisdiction, courts typically referred to the ‘benefit’ of the individual: see L Shelford, *Practical Treatise on the Law concerning Lunatics, Idiots and Persons of Unsound Mind* (J.S. Little 1827). It was only in the wake of *Re F (Mental Patient: Sterilisation)* [1990] 2 AC 1 that the term was imported (without any consideration of alternatives) from the exercise of judicial discretion in respect of minors as the applicable standard for the newly recognised declaratory jurisdiction.

92 See R Mnookin, ‘Child Custody Adjudication: Judicial Functions in the Face of Indeterminacy’ (1975) 39 Law & Contemp Probs 226.

93 On the enhanced role of intuitions in situations where decision-makers rely on shorthand, see A Tversky and D Kahneman, ‘Judgment under Uncertainty: Heuristics and Biases’ (1974) 185 Science 1124; C Sunstein, ‘Moral Heuristics and Moral Framing’ (2003–2004) 88 Minn L Rev 1556.

94 The Assisted Decision-Making (Capacity) Act 2015, s 8 sets out eleven applicable principles (and several sub-principles), most of which are identical to the principles set out in MCA, s 1 and the components of the best interests standard in MCA, s 4.

95 This is evident in the requirement that an intervener must ‘act at all times in good faith and for the benefit of the relevant person’: s 8(7)(e).

96 [2014] EWCOP B9, § 10.

